# The Absence of Extracellular Cold-Inducible RNA-Binding Protein (eCIRP) Promotes Pro-Angiogenic Microenvironmental Conditions and Angiogenesis in Muscle Tissue Ischemia

**DOI:** 10.3390/ijms22179484

**Published:** 2021-08-31

**Authors:** Matthias Kübler, Sebastian Beck, Lisa Lilian Peffenköver, Philipp Götz, Hellen Ishikawa-Ankerhold, Klaus T. Preissner, Silvia Fischer, Manuel Lasch, Elisabeth Deindl

**Affiliations:** 1 Walter-Brendel-Centre of Experimental Medicine, University Hospital, Ludwig-Maximilians-Universität München, 81377 Munich, Germany; Matthias.Kuebler@med.uni-muenchen.de (M.K.); sebastian.beck@med.uni-muenchen.de (S.B.); P.Goetz@med.uni-muenchen.de (P.G.); Hellen.Ishikawa-Ankerhold@med.uni-muenchen.de (H.I.-A.); manuel_lasch@gmx.de (M.L.); 2 Biomedical Center, Institute of Cardiovascular Physiology and Pathophysiology, Ludwig- Maximilians-Universität München, 82152 Planegg-Martinsried, Germany; 3 Department of Biochemistry, Faculty of Medicine, Justus Liebig University, 35392 Giessen, Germany; Lilli.Peffenkoever@gmail.com (L.L.P.); Klaus.T.Preissner@biochemie.med.uni-giessen.de (K.T.P.); Silvia.Fischer@biochemie.med.uni-giessen.de (S.F.); 4 Department of Internal Medicine I, Faculty of Medicine, University Hospital, Ludwig-Maximilians-Universität München, 81377 Munich, Germany; 5 Department of Otorhinolaryngology, Head and Neck Surgery, University Hospital, Ludwig-Maximilians-Universität München, 81377 Munich, Germany

**Keywords:** angiogenesis, cold-inducible RNA-binding protein, extracellular cold-inducible RNA-binding protein, CIRP, eCIRP, neutrophil extracellular traps, NETs, macrophage polarization, inflammation, apoptosis, ischemia

## Abstract

Extracellular Cold-inducible RNA-binding protein (eCIRP), a damage-associated molecular pattern, is released from cells upon hypoxia and cold-stress. The overall absence of extra- and intracellular CIRP is associated with increased angiogenesis, most likely induced through influencing leukocyte accumulation. The aim of the present study was to specifically characterize the role of eCIRP in ischemia-induced angiogenesis together with the associated leukocyte recruitment. For analyzing eCIRPs impact, we induced muscle ischemia via femoral artery ligation (FAL) in mice in the presence or absence of an anti-CIRP antibody and isolated the gastrocnemius muscle for immunohistological analyses. Upon eCIRP-depletion, mice showed increased capillary/muscle fiber ratio and numbers of proliferating endothelial cells (CD31^+^/CD45^−^/BrdU^+^). This was accompanied by a reduction of total leukocyte count (CD45^+^), neutrophils (MPO^+^), neutrophil extracellular traps (NETs) (MPO^+^CitH3^+^), apoptotic area (ascertained via TUNEL assay), and pro-inflammatory M1-like polarized macrophages (CD68^+^/MRC1^−^) in ischemic muscle tissue. Conversely, the number of regenerative M2-like polarized macrophages (CD68^+^/MRC1^+^) was elevated. Altogether, we observed that eCIRP depletion similarly affected angiogenesis and leukocyte recruitment as described for the overall absence of CIRP. Thus, we propose that eCIRP is mainly responsible for modulating angiogenesis via promoting pro-angiogenic microenvironmental conditions in muscle ischemia.

## 1. Introduction

The cold-inducible RNA binding protein (CIRP) is a member of the glycine-rich RNA-binding protein family, including several proteins which regulate nucleic acid interactions through their RNA-binding sites [1,2]. The expression of intracellular CIRP (iCIRP) in various cell types, particularly in immune cells, is induced in response to diverse cellular stresses such as mild hypothermia, hypoxia, or oxidative stress, and in response, the intracellular protein can be translocated from the nucleus to the cytoplasm [1,3,4,5,6,7]. In particular, as a reaction to hypoxia, in hemorrhagic shock and other ischemia-related pathologies, iCIRP translocates from the cytoplasm towards the extracellular space (possibly via lysosomal secretion) [8,9,10,11,12]. Once secreted, extracellular CIRP (eCIRP) acts as a damage-associated molecular pattern (DAMP) molecule, leading to tissue damage and aggravation of inflammation [8]. eCIRP activates a wide range of immune cells, such as neutrophils and macrophages, via the “Toll-like receptor 4” (TLR4)-myeloid differentiation factor 2 (MD2)-complex as well as via the “Triggering receptor expressed on myeloid cells 1” (TREM-1). These interactions result in amplified inflammatory processes through the release of pro-inflammatory cytokines and chemokines [8,13,14].

The absence of eCIRP in most of the studied ischemic disease-related animal models was correlated with a significant mitigation of the investigated inflammatory reaction, while the injection of recombinant CIRP led to liver and lung injury [10,15,16,17,18,19]. Furthermore, patients brought to the intensive care unit due to hemorrhagic shock or sepsis presented with a significantly higher survival chance when serum levels of CIRP were low [8,20]. Hence, the modulation of eCIRP’s bioavailability may provide a novel approach in therapeutic drug treatment in the immune regulation of different inflammatory-dependent pathologies.

Several RNA-binding proteins (RBP) have been implicated in the post-transcriptional regulation of mRNAs involved in the processes of angiogenesis [21,22,23]. Angiogenesis describes the generation of new capillaries from a pre-existing capillary network, particularly in response to ischemia, necessary for embryonic development as well as for wound healing, menstruation, or pregnancy in healthy adults [24,25,26,27]. The predominant pro-angiogenic cytokine involved in these processes is vascular endothelial growth factor A (VEGF-A) [24,28,29], whose expression is induced in ischemic tissues under the control of a molecular oxygen-sensor, present in virtually all cell types [30,31]. Following endothelial cell activation, extracellular matrix remodeling by proteases and anastomosis formation of capillary beds, regulatory humoral factors, as well as immune cells (particularly macrophages) contribute to the orchestration and control of angiogenesis [24].

To increase the capillarity of an ischemic tissue, there are two different mechanisms through which angiogenesis can proceed: (a) splitting the capillaries of a pre-existing microvasculature network, a process which is denoted as intussusceptive angiogenesis; (b) de novo formation of new capillary branches by migration and proliferation of endothelial cells, a mechanism that is referred to as sprouting angiogenesis [25,32,33]. Besides physiological angiogenesis, uncontrolled pathological angiogenesis drives solid tumor growth, psoriasis, or diabetic retinopathy [24,26,27,34,35,36], where the indicated factors appear to operate in an uncontrolled manner. Concerning vascular diseases, like myocardial infarction or peripheral artery disease, the process of angiogenesis alone is not sufficient to restore the blood supply in the affected ischemic areas [37]. In this case, capillary growth is promoted to clear the cell debris congregated at the site of ischemia. Instead, only the process of arteriogenesis, the growth transformation of pre-existing vessels acting as natural bypasses in response to increased shear-stress, can compensate for the occlusion of a nutrient supplying artery [38,39,40,41,42].

Particularly myeloid cells, such as neutrophils and macrophages, but also blood platelets, are major sources of VEGF-A and other vascular remodeling factors like matrix metalloproteinases (MMPs) that regulate the bioavailability of VEGF-A and participating in vessel formation and the clearance of cell debris at the ischemic tissue site [43,44,45,46,47]. As a consequence, the accumulation and activation of different leukocyte subpopulations in certain vascular regions may directly influence the outcome of angiogenic vessel formation.

Neutrophil extracellular traps (NETs), comprised of the decondensed extracellular chromatin network of activated neutrophils, and necessary for catching and killing of microbes in innate immunity, were found to positively affect angiogenesis in a preclinical model of pulmonary hypertension through the upregulation of intercellular adhesion molecule 1 (ICAM-1) expression, affecting VEGF-signaling and endothelial cell migration [48].

Recently, a new subset of pro-inflammatory neutrophils that could be triggered by eCIRP under septic conditions were shown to express high levels of C-X-X chemokine receptor type 4 (CXCR4), ICAM-1, inducible nitric oxide synthase (iNOS), reactive oxygen species (ROS), and NETs [49]. Furthermore, eCIRP was described to excessively induce NET formation by activating the “Triggering receptor expressed on myeloid cells 1” (TREM-1) in a Rho-GTPase-dependent manner and via the upregulated expression of “Peptidylarginine deiminase 4” (PAD4), which catalyzes the citrullination of histones as an essential step in NET formation to decondense the cellular chromatin [50,51,52,53].

Idrovo et al. (2016) found a significant improvement of wound healing of skin lesions in CIRP-deficient mice as compared to wild-type mice. The elevated inflammatory state (such as changes in tumor necrosis factor-α (TNF-α) dynamics, reduced numbers of Gr1^+^ leukocytes) was accompanied by an elevated number of CD31^+^ cells, resulting in the hypothesis that the absence of CIRP could improve tissue regeneration and angiogenesis [54]. Moreover, iCIRP was suggested to regulate the post-transcriptional processing of specific microRNAs of the 14q32 locus, which are known to be involved in regulating ischemia-induced angiogenesis [55,56]. It was shown that the blocking of miR-329, a microRNA belonging to the 14q32 cluster and a possible target of post-transcriptional processing of CIRP, enhanced the expression of CD146, a co-receptor of VEGF receptor 2 (VEGFR-2) on endothelial cells, also to ameliorate angiogenic processes [56,57,58].

Recently, we showed in a murine hindlimb model that the genetic ablation of CIRP (including eCIRP and iCIRP) improves angiogenesis and the regeneration of ischemic tissue damage, most likely through the predominance of regenerative anti-inflammatory M2-like polarized macrophages and the reduced accumulation of neutrophils and NETs [59]. Whether these changes in leukocyte recruitment, macrophage polarization, and ameliorated angiogenesis are attributed to the lack of intra- or extracellular CIRP remains unclear so far. In the present study, we have analyzed the ramifications of blocking eCIRP on ischemia-induced angiogenesis and cell apoptosis together with the accompanied leukocyte infiltration and macrophage polarization in order to demonstrate that eCIRP appears to be sufficient to modulate the efficacy of angiogenesis in vivo.

## 2. Results

To analyze the impact of the lack of eCIRP on the process of angiogenesis in ischemic muscle tissue, a well-established murine hindlimb model was used. Following femoral artery ligation (FAL), the formation of collateral blood vessels (arteriogenesis) is initiated in the adductor muscle of the upper leg, and in ischemia-induced angiogenesis in the gastrocnemius muscle of the lower leg, due to the reduced blood flow [60]. After intravenous injection of mice with a neutralizing anti-CIRP antibody prior to and following FAL (every second day) [9], gastrocnemius muscles were collected for immunohistological analyses at day 1 and day 7 after FAL. An isotype antibody or phosphate-buffered saline (PBS), respectively, was used in the control groups.

**Figure 1:**
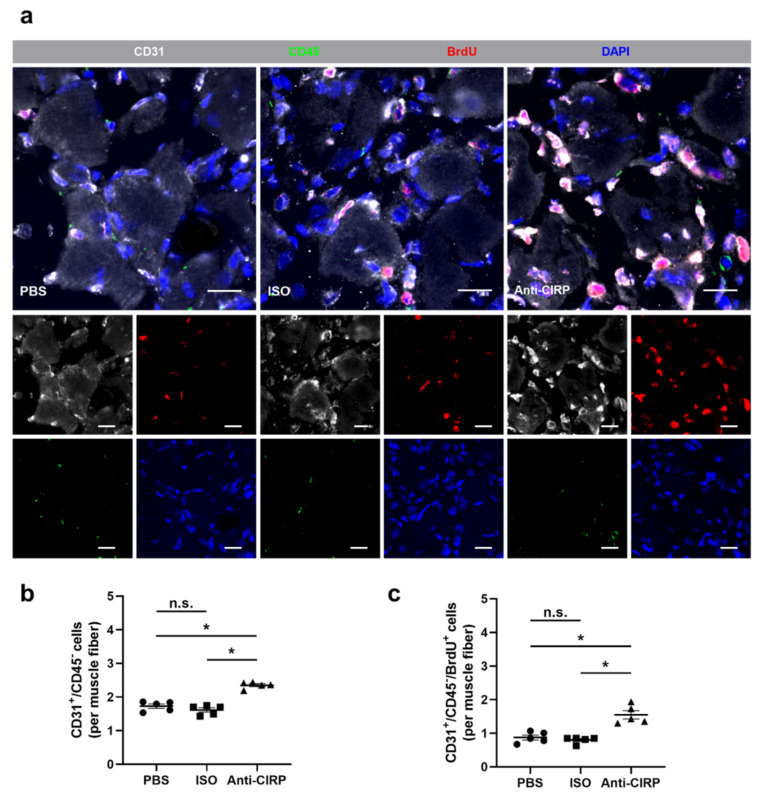
Lack of extracellular “Cold-inducible RNA-binding protein” (eCIRP) enhances capillary growth. (**a**) Representative immunofluorescence stains of ischemic gastrocnemius muscle slices from control mice (treated with phosphate-buffeCDred saline (PBS) or isotype antibody (ISO)), and mice which received anti-CIRP antibody (Anti-CIRP) 7 days after femoral artery ligation (FAL). Smaller images show single channels, large images show all merged channels of endothelial cells (anti-CD31, white), proliferating cells (anti-BrdU (bromodeoxyuridine), red), leukocytes (anti-CD45, green), and nucleic acid (DAPI, blue). Scale bars represent 20 µm. Scatter plots displaying (**b**) CD31^+^/CD45^−^ (endothelial) cells and (**c**) CD31^+^/CD45^−^/BrdU^+^ (proliferating endothelial) cells per muscle fiber of ischemic gastrocnemius muscles of PBS-, ISO- (both control groups), and anti-CIRP antibody-treated mice 7 days after FAL. Data are means ± S.E.M., n.s. *p* > 0.05, * *p* < 0.05 (PBS vs. ISO vs. anti-CIRP) by one-way ANOVA with the Tukey’s multiple comparisons test, a defined ischemic area (1.5 mm^2^) of muscle tissue was analyzed per mouse.

In order to investigate whether the depletion of eCIRP affects angiogenesis, a CD31/CD45/BrdU/DAPI quadruple immunofluorescence staining on tissue sections collected 7 days after FAL was performed. CD31 was implemented as an endothelial cell marker, CD45 was used as a pan-leukocyte marker to exclude CD31^+^ leukocytes. To exclude platelets from the quantification (which also express CD31), only CD31^+^ cells that colocalized with a signal for nuclear DNA (DAPI) were counted. Hence, CD31^+^/CD45^−^/DAPI^+^ cells were defined as capillary endothelial cells. Bromodeoxyuridine (BrdU) was used as a proliferation marker. To quantitate the extent of angiogenesis, the number of capillaries per muscle fiber ratio was determined [61]. Compared to both control groups (isotype antibody-and PBS-treated mice), the anti-CIRP antibody-treated group showed a significant increase in capillary/muscle fiber ratio (Figure 1a,b). Furthermore, an elevated ratio of proliferating capillaries (CD31^+^/CD45^−^/BrdU^+^/DAPI^+^) per muscle fiber was found in anti-CIRP antibody-treated mice compared to both control groups (Figure 1a,c). Isotype antibody- and PBS-treated mice did not show any statistically significant difference between their capillarity and proliferating endothelial cells per muscle fiber. In tissue samples of non-ischemic (sham-operated) gastrocnemius muscles no significant difference in capillary per muscle fiber ratio between all treatment groups was noted (data not shown).

Upon treatment of microvascular endothelial cells in vitro with recombinant murine CIRP (rmCIRP), a significant reduction in fetal calf serum (FCS)-initiated cell proliferation was seen (Figure S1). Moreover, after rmCIRP treatment of cells, the mRNA levels of the inflammatory genes interleukin 6 (IL-6) and monocyte chemoattractant protein-1 (MCP-1) were significantly elevated, whereas the expression level of CIRP did not change (Figure S2).

**Figure 2:**
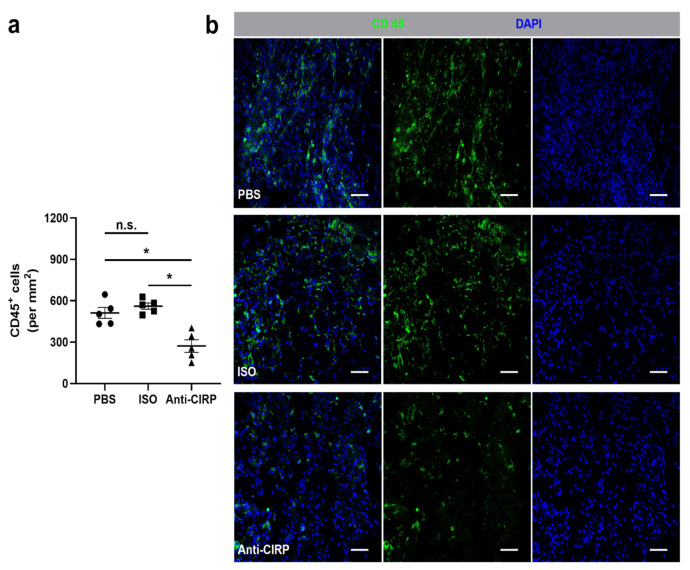
Neutralization of extracellular “Cold-inducible RNA-binding protein” (eCIRP) decreases leukocyte accumulation in ischemic tissue. (**a**) The scatter plot displays the relative number of CD45^+^ (pan-leukocyte marker) cells (per mm^2^) in the ischemic gastrocnemius muscles of mice that received phosphate-buffered saline (PBS), isotype antibody (ISO) (control groups), or the anti-CIRP antibody (Anti-CIRP) and were sacrificed 7 days after femoral artery ligation (FAL). Data are means ± S.E.M., *n* = 5 per group. n.s. *p* > 0.05, * *p* < 0.05 (PBS vs. ISO vs. Anti-CIRP) by one-way ANOVA with the Tukey’s multiple comparisons test, a defined ischemic area (1.5 mm^2^) of muscle tissue was analyzed per mouse. (**b**) Representative immunofluorescence staining of ischemic gastrocnemius muscles of mice treated with PBS (top), isotype antibody (middle), or anti-CIRP antibody (bottom) 7 days after FAL. Cells were stained with an antibody against CD45 (green) and DAPI (blue) to label nucleic DNA. Scale bars represent 50 µm.

Leukocytes, such as neutrophils and macrophages, play a regulatory role in ischemia-induced inflammation through the removal of cellular debris, the promotion of tissue repair, and their direct influence on vascular proliferation by supplying a broad range of growth factors. In response to the neutralization of eCIRP in the anti-CIRP antibody-treated mice on day 7 after FAL, the number of infiltrated CD45^+^ cells in ischemic areas was significantly decreased as compared to both control groups (Figure 2a,b). In all non-ischemic (sham-operated) muscle tissues of each group, no difference in the numbers of infiltrated CD45^+^ cells was found (data not shown).

**Figure 3:**
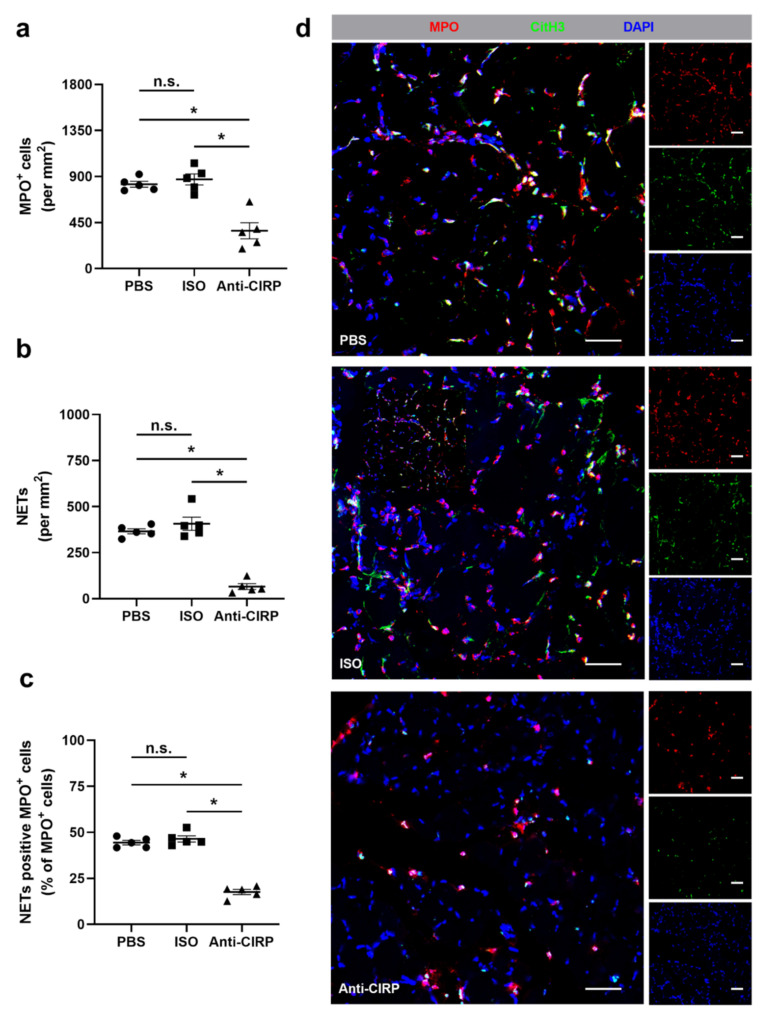
Depletion of extracellular “Cold-inducible RNA-binding protein” (eCIRP) interferes with neutrophil recruitment and neutrophil extracellular trap (NET) formation in ischemic muscle tissue. The scatter plots display the number of (**a**) MPO^+^ (myeloperoxidase, marker for neutrophils) cells, (**b**) NETs (MPO^+^/CitH3^+^ (citrullinated histone 3)/DAPI^+^) (both per mm^2^), and (**c**) NET positive MPO^+^ cells per total MPO^+^ cells in ischemic gastrocnemius muscles isolated from phosphate-buffered saline- (PBS), isotype antibody- (ISO) (control groups) and anti-CIRP antibody- (Anti-CIRP) treated mice, isolated on day 1 after femoral artery ligation (FAL). Data are means ± S.E.M., n = 5 per group. n.s. *p* > 0.05, * *p* < 0.05 (PBS vs. ISO vs. Anti-CIRP) by one-way ANOVA with the Tukey’s multiple comparisons test, a defined ischemic area (1.5 mm^2^) of muscle tissue was analyzed per mouse. (**d**) Representative immunofluorescence staining of ischemic gastrocnemius muscle slices of PBS- (top), isotype antibody- (middle), and anti-CIRP antibody-treated mice (bottom) collected 1 day after FAL. Images display single and merged channels of neutrophils (MPO^+^/DAPI^+^) and NETs (MPO^+^/CitH3^+^/DAPI^+^) labeled with anti-MPO (red), anti-CitH3 (green), and DAPI (nucleic acid, blue). Scale bars represent 50 µm.

In order to assess the influence of eCIRP neutralization on the accumulation of different neutrophil subpopulations and their specific products (such as NETs) in ischemic muscle tissue sections, double immuno-staining at day 1 after FAL for myeloperoxidase (MPO) (to detect neutrophils) and for citrullinated histone H3 (CitH3) (to detect NETs) was performed. MPO^+^/DAPI^+^ cells were classified as neutrophils and MPO^+^/ CitH3^+^/DAPI^+^ cells were considered as NETs. A significantly reduced number of both, neutrophils and NETs, was seen in tissue samples of mice treated with anti-CIRP antibody compared to the two control groups (Figure 3a,b,d). Also, the portion of neutrophils forming NETs in comparison to the total neutrophil count was significantly reduced in anti-CIRP antibody-treated mice (Figure 3c,d). No differences were observed between all control groups in non-ischemic (sham-operated) muscle tissue sections (data not shown).

**Figure 4:**
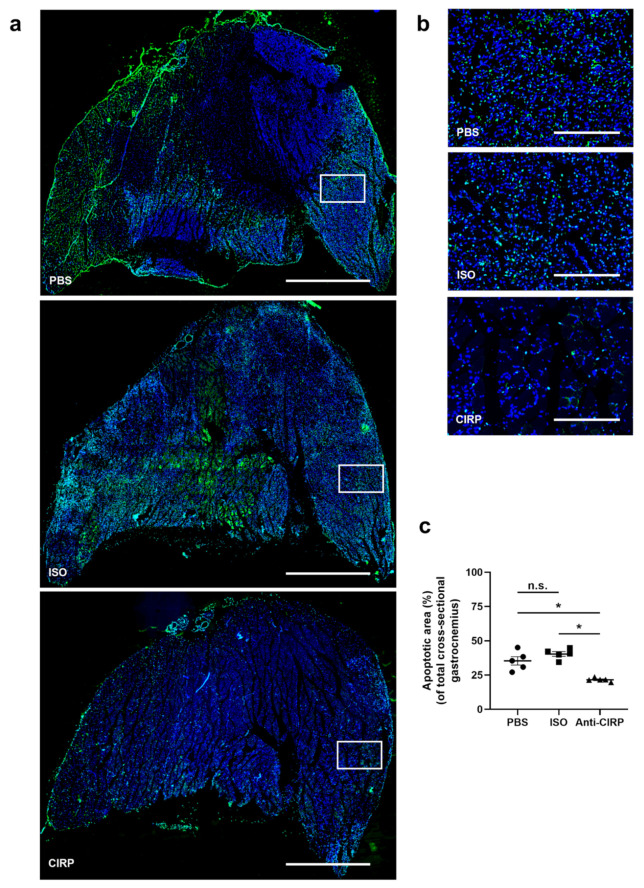
Depletion of extracellular “Cold-inducible RNA-binding protein” (eCIRP) mitigates apoptosis. (**a**) Representative pictures of TUNEL-stained gastrocnemius muscle slices of mice, treated with phosphate-buffered saline (PBS) (top), isotype antibody (ISO) (middle) (both control groups), and anti-CIRP antibody (Anti-CIRP) (bottom) 7 days after femoral artery ligation (FAL). Scale bars represent 50 µm. (**b**) Magnification of white boxes in (**a**), showing TUNEL-stained apoptotic cells (green). Scale bars represent 7 µm. (**c**) The scatter plot displays the extent of the apoptotic areas in relation to the whole gastrocnemius muscle of PBS-, ISO-, and anti-CIRP antibody-treated mice 7 days after FAL. The total gastrocnemius cross-sectional area (about 20 mm^2^) was analyzed. Data are means ± S.E.M., *n* = 5 per group. n.s. *p* > 0.05, * *p* < 0.05 (PBS vs. ISO vs. Anti-CIRP) by one-way ANOVA with the Tukey’s multiple comparisons test.

Based on the observation that eCIRP-induced NETs have an impact on efferocytotic clearance of apoptotic cells by macrophages [62], the extent of apoptotosis in ischemic muscle tissue sections 7 days after FAL was assessed by using a TdT-mediated dUTP-biotin neck end labeling (TUNEL) assay. A significant reduction of the apoptotic area in gastrocnemius muscle sections from the anti-CIRP antibody-treated group was found compared to the isotype antibody-treated and PBS-treated control groups, respectively (Figure 4a–c). There was no sign of apoptosis in the isolated non-ischemic (sham-operated) gastrocnemius muscles from all groups (data not shown).

**Figure 5:**
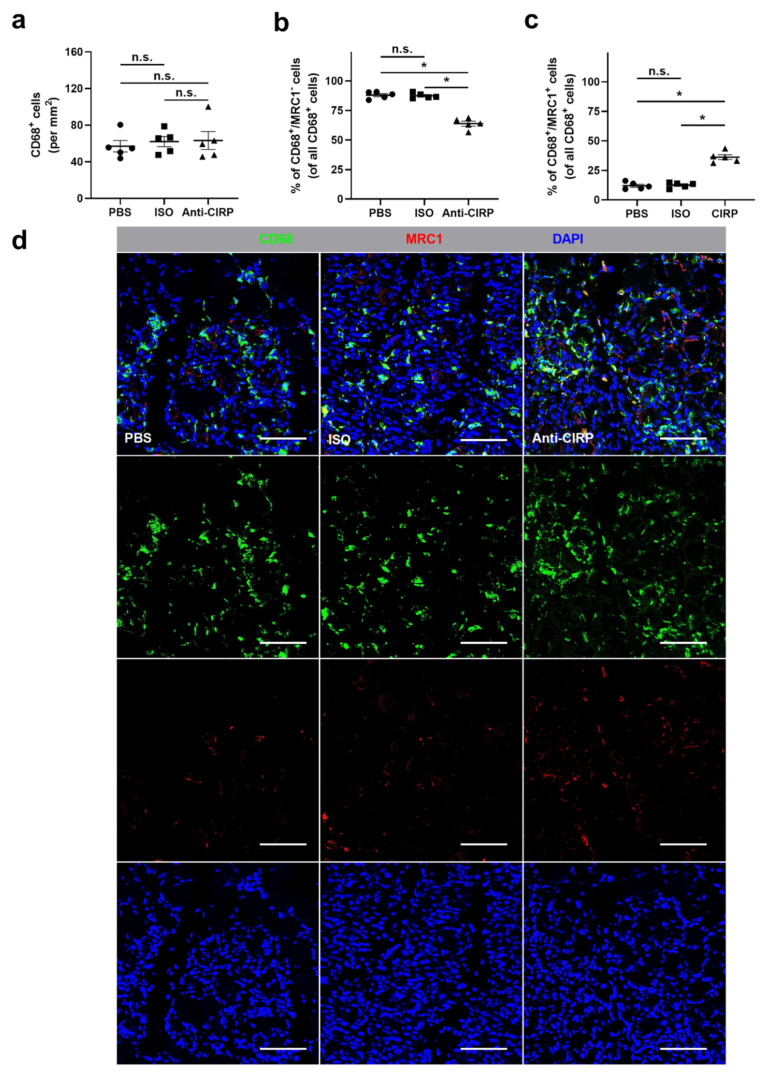
Neutralization of extracellular “Cold-inducible RNA-binding protein” (eCIRP) affects macrophage polarization. The scatter plots display (**a**) the relative amount of CD68^+^ cells (macrophages) (per mm^2^), (**b**) CD68^+^/MRC1^−^ (mannose receptor c-type 1) cells (M1-like polarized macrophages), and (**c**) CD68^+^/MRC1^+^ cells (M2-like polarized macrophages) in relation to all CD68^+^ cells (in percent) in ischemic gastrocnemius muscles of phosphate-buffered saline- (PBS), isotype antibody- (ISO) (both control groups), or anti-CIRP antibody-treated mice (Anti-CIRP) 7 days after femoral artery ligation (FAL). Data are means ± S.E.M., n = 5 per group. n.s. *p* > 0.05, * *p* < 0.05 (PBS vs. ISO vs. Anti-CIRP) by one-way ANOVA with the Tukey’s multiple comparisons test, a defined ischemic area (1.5 mm^2^) of muscle tissue was analyzed per mouse. (**d**) Representative immunofluorescence staining of ischemic gastrocnemius muscle slices of PBS- (left), isotype antibody- (middle), and anti-CIRP-antibody-treated mice (right) 7 days after FAL. Images show single and merged channels of CD68 and MRC1 labeled macrophages (anti-CD68, green; anti-MRC1, red) and nucleic acid (DAPI, blue). Scale bars represent 50 µm.

The influence of the neutralization of eCIRP on macrophage accumulation and polarization was analyzed by using anti-CD68 antibody staining to label macrophages as well as anti-MRC1 (mannose receptor C-type 1) antibody to identify M2-like polarized macrophages. Consequently, CD68^+^/MRC1^−^/DAPI^+^ cells were counted as M1-like polarized macrophages, while CD68^+^/MRC1^+^/DAPI^+^ cells were assessed as M2-like polarized macrophages. No significant differences in macrophage accumulation at the site of ischemia of gastrocnemius muscles, collected 7 days after FAL, were observed between all three different treatment groups (Figure 5a,d). However, a significantly higher portion of M2-like polarized macrophages and a significant reduction in the portion of M1-like polarized macrophages in the ischemic muscle tissue in anti-CIRP antibody-treated mice was found (Figure 5b–d). Gastrocnemius muscles of all experimental groups isolated from the contralateral sham-operated (non-ischemic) site did not show any significant difference in macrophage accumulation and polarization (data not shown).

## 3. Discussion

**Figure 6:**
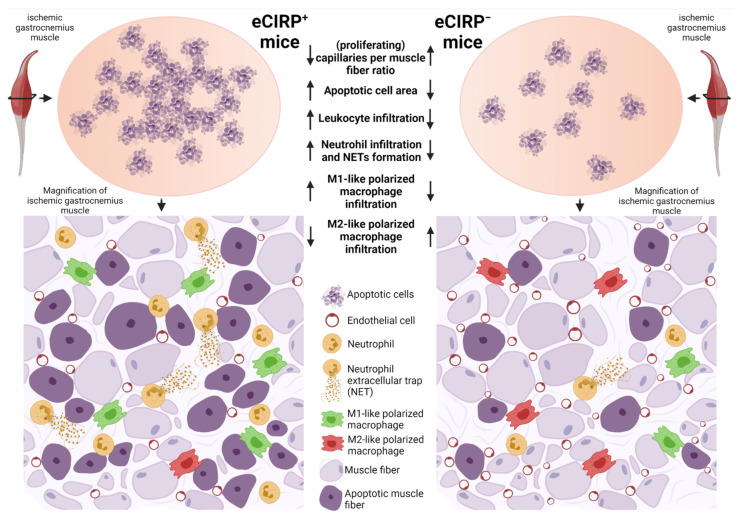
Depletion of extracellular “Cold-inducible RNA-binding protein” (eCIRP) promotes ischemia-induced angiogenesis and affects the associated leukocyte infiltration. In comparison to control mice, which still harbor eCIRP, mice with neutralized eCIRP showed an increased (proliferating) capillaries per muscle fiber ratio, reduced areas of apoptotic cells, reduced leukocyte infiltration with lower numbers of neutrophils, neutrophils that produce neutrophil extracellular traps (NETs), and pro-inflammatory M1-like polarized macrophages, whereas the number of infiltrative anti-inflammatory regenerative M2-like polarized macrophages was elevated. Thus, eCIRP-depleted mice show an ameliorated angiogenic capacity upon ischemia, mediated through a more pro-angiogenic inflammatory environment.

In the current study, the impact of the neutralization of extracellular CIRP (eCIRP) on angiogenesis and the associated leukocyte accumulation in a murine hindlimb model of muscle ischemia was investigated. The depletion of eCIRP resulted in ameliorated angiogenesis, evidenced by an increased capillary to muscle fiber ratio, as well as a reduction of the total apoptotic area in gastrocnemius muscle of mice treated with an anti-CIRP antibody. These responses might be due to a significant reduction in leukocyte accumulation, particularly neutrophil infiltration and NET formation, and a pronounced influence on macrophage polarization, although the number of infiltrated macrophages was not altered in ischemic gastrocnemius. In anti-CIRP antibody-treated mice a predominance of the anti-inflammatory M2-like polarized macrophages at the ischemic muscle tissue site was observed, as compared to control mice (for an overview see Figure 6). Overall, the blockade of eCIRP enhanced angiogenesis in vivo, reminiscent of results obtained in CIRP-deficient mice [59], to indicate that eCIRP is mainly responsible for modulating angiogenesis.

It has been described that the deficiency of CIRP in mice did affect angiogenesis in association with different pathologies [54,56]. We recently demonstrated that the overall absence of CIRP, intra- and extracellularly, resulted in ameliorated angiogenesis in muscle ischemia [59]. As evidenced by immunohistological analysis, the present study now shows that the blockade of eCIRP resulted in an elevated capillary to muscle fiber ratio and a raised proliferating capillary to muscle fiber ratio, thus improving angiogenesis. Furthermore, we confirmed our in vivo findings, as the administration of rmCIRP on microvascular endothelial cells abolished their FCS-induced proliferation in vitro. Consequently, for the first time it is shown that the neutralization of eCIRP by the administration of a specific antibody in the indicated mouse model resulted in ameliorated angiogenesis. Hence, the improved angiogenesis in CIRP-deficient mice is largely attributable to their lack of eCIRP.

Since connections between eCIRP as DAMP and immune cells are well described [63,64], we evaluated the influence of eCIRP depletion on leukocyte accumulation at the site of the ischemic gastrocnemius muscle tissue. In fact, a significant reduction in leukocyte accumulation, particularly neutrophils, at the site of muscle ischemia was observed when eCIRP was blocked.

In the context of angiogenesis and tissue reorganization, infiltrative leukocytes, predominantly neutrophils and macrophages, are known to be essential sources of pro-angiogenic growth factors, including VEGF-A, and several proteases, such as matrix metalloproteinase 9 (MMP9) which are important modulators of the extracellular matrix remodeling [43,47,65,66,67]. The diminished numbers of leukocytes are likely attributable to the lack of eCIRP as an inflammatory DAMP. eCIRP activates both macrophages and neutrophils via its direct binding to the pattern recognition receptor (PRR) TLR4-MD2-complex, resulting in nuclear factor ‘kappa-light-chain-enhancer’ of activated B-cells (NF-κB) activation and nuclear translocation, or to the TREM-1, thereby activating the tyrosine kinase Syk to ultimately catalyze the release of pro-inflammatory chemokines and cytokines, relevant for the exacerbation of inflammation and the enhancement of leukocyte recruitment [8,13,14,68]. Leukocyte accumulation and transmigration into the damaged tissue might be further facilitated by eCIRP´s propensity to increase vascular permeability and to activate endothelial cells, causing additional pro-inflammatory chemoattractant release and an upregulation of cell-surface adhesion molecules like endothelial-selectin (E-selectin) and ICAM-1 [19]. In addition, the administration of rmCIRP was shown to strongly induce the expression of inflammatory genes in microvascular endothelial cells in vitro. Consequently, the blocking of eCIRP could be responsible for a reduced accumulation of leukocytes at the site of the ischemic muscle tissue due to the absence of eCIRP´s pro-inflammatory properties as a DAMP and an attenuated extravasation induced by a more stable endothelial cell barrier and a decreased upregulation of cell-surface adhesion molecules.

It is important to mention that an excessive and prolonged accumulation of leukocytes at the site of inflammation can lead to aggravated tissue damage and thus may interfere with ischemic tissue restitution [47,65,69]. The blockade of eCIRP in many other pathologies had an attenuating influence on the inflammation in animal models, while low eCIRP levels in septic patients correlated with improved survival chances. By comparing the ischemic muscle tissue of CIRP-deficient and wild-type mice, we found a significant reduction in leukocyte accumulation in the CIRP knockout mice [59]. Accordingly, we propose that the decreased leukocyte infiltration in CIRP-deficient mice is mainly attributed to the lack of eCIRP. Whether iCIRP plays a role in leukocyte accumulation and its affection on immune cells in general, possibly through miRNA interactions or via binding of intracellular PRRs like nucleotide-binding, oligomerization domain (NOD)-like receptors (NLRs) or the RIG-like helicases (RLHs), must be elucidated in further in-depth immunology studies.

In the early phase of tissue damage, neutrophils infiltrate the ischemic tissue as one of the first innate immune cell types, playing an essential role in orchestrating angiogenesis and inflammation [66]. Comparable to macrophages, they are also capable of phagocyting apoptotic cells, thus clearing cell debris from the damaged tissue areas and consequently participating in tissue homeostasis restitution. Particularly important, neutrophils significantly influence the initiation of angiogenesis, since neutropenic mice showed impaired induction of angiogenesis [70]. Most likely, neutrophils participate in angiogenesis initiation through their direct allocation of preformed VEGF-A and other growth factors [44,45]. Their supply of active MMP9 and the protease-associated deconstruction of the extracellular matrix leads to further release of former matrix-bound VEGF-A and consequently to angiogenic sprouting [46,71]. Besides delivering VEGF-A to the ischemic site, activated neutrophils release a wide range of pro-inflammatory cytokines and chemokines, leading to the disruption of the endothelial cell barrier and further leukocyte recruitment, ultimately exacerbating the inflammation in damaged tissue [72]. For a long time, neutrophils were thought to be the major cause for aggravated and prolonged muscle injury [73]. Recent studies discounted this by proving that the blockade of neutrophils interfered with tissue restoration processes in muscle injury [74,75]. Hence, neutrophil-associated damage in muscle tissue could be substantial for recovery processes. After clearing cell debris, modulating inflammation, and initiating angiogenesis, neutrophils do not get phagocyted by macrophages but may reenter the vasculature by a process declared as reverse transendothelial migration [76].

Although neutrophils are important players in initiating angiogenesis, in our study, we found reduced numbers of neutrophils in eCIRP-depleted mice which, however, exhibited increased capillarity. We made similar observations in CIRP-knockout mice [59]. Furthermore, decreased neutrophil recruitment in anti-CIRP antibody-treated mice was also observed in a model of hepatic ischemia and reperfusion injury [9]. In our study, the diminished number of accumulated neutrophils observed 24 h after FAL in eCIRP-depleted mice might not be related to an overall reduction of neutrophil infiltration at all. It could reflect an augmented reverse transendothelial migration after phagocytosis of cell debris, initiation of angiogenesis, culminating in the orchestration of inflammation. Interestingly, under septic conditions, eCIRP was found to induce reverse transendothelial migration [77]. Yet, in contrast to sterile inflammation, reverse transendothelial migration of neutrophils in sepsis does not contribute to tissue reconstitution. Instead, neutrophils reentering the circulation in sepsis may further fuel inflammatory reactions and lead to dissemination of a local towards a systemic inflammation [77]. Whether the increased number of neutrophils in control mice that harbor eCIRP rather reflects an unrestrained inflammation as opposed to the effective induction of angiogenesis and whether the lack of eCIRP possibly ameliorates neutrophil reverse transendothelial migration in ischemia-dependent tissue damage are two possible processes that deserve further analysis.

In the present study, we found a significant reduction of NETs and NET-forming neutrophils in mice treated with the anti-CIRP antibody compared to the control groups. These observations are in line with previous findings, showing that DAMPs in general and eCIRP in particular are potent inducers of NET formation [50,51,52,78]. Interestingly, eCIRP promotes the induction of a specific pro-inflammatory subtype of neutrophils, expressing ICAM-1, and are characterized among others through their increased formation of NETs [52]. Moreover, we found decreased NET formation in ischemic muscle tissue of CIRP-deficient mice as well [59]. Whether NETs released by pro-inflammatory ICAM-1^+^ neutrophils have the same effect on angiogenesis needs to be addressed in further investigations. Here, it is important to mention that NETs have been described not only to be associated with ongoing inflammation, angiogenesis, and vascular regeneration but are also causative for aggravated inflammation and thus evoke exacerbated tissue damage and delayed tissue restitution [79,80,81,82,83]. Therefore, we propose that the depletion of eCIRP may promote a more pro-angiogenic type of NET formation, contributing to tissue remodeling in the ischemic mice muscle.

A recent study on sepsis showed that NET formation, caused by eCIRP, limited efferocytosis [62]. Efferocytosis describes the process by which apoptotic cells are cleared by phagocytic cells, such as macrophages, and provides a prerequisite for resolving inflammation [84]. Efferocytotic clearance of apoptotic cells stimulates anti-inflammatory and pro-regenerating signals, marking the start of the tissue remodeling phase [84]. To ascertain whether abated efferocytosis also affected apoptotic processes in our study, we implemented a TUNEL assay to measure the area of apoptotic cells throughout the entire sections of the gastrocnemius muscle. The observation that reduced apoptotic areas in eCIRP-depleted mice were found may reflect an improved efferocytosis in these animals. Accordingly, excessive NET formation in control mice would not promote angiogenesis but would prolong the inflammatory phase leading to enhanced leukocyte recruitment. In contrast, apoptotic areas in eCIRP-depleted mice would be cleared much faster and could lead to an earlier start of the subsequent tissue remodeling phase. Whether the diminished areas of apoptotic cell death in the ischemic muscles of eCIRP-depleted mice are a direct consequence of eCIRP´s effect on efferocytosis or are caused by another independent mechanism needs to be elucidated.

After the initial inflammatory phase, mainly characterized by the infiltration of neutrophils, macrophages accumulate at the site of ischemic muscle tissue damage, and have a significant impact on angiogenesis [24,47,85,86,87]. It is important to note that macrophages are a heterogeneous cell population, presenting high plasticity, reflected by their variable polarization states [88,89]. Therefore, the M1- and M2-like polarization status classification only marks extremes in a broad spectrum of possible differentiations.

Initially, M0-like polarized monocytes infiltrate the damaged tissue and locally mature to classically activated pro-inflammatory M1-like (CD68^+^/MRC1^−^) polarized macrophages. Macrophages showing this pro-inflammatory polarization state are responsible for phagocytosis and further leukocyte recruitment. In terms of angiogenesis, macrophages representing the M1-like polarized phenotype are associated with the supply of pro-angiogenic factors, such as VEGF-A and TNF-α, thus being relevant for the induction of angiogenesis [90]. It has been shown that M1-like polarized macrophages cumulate around endothelial tip cells, guiding the new sprouts, whereas the absence of pro-inflammatory macrophages resulted in the thwarted instigation of angiogenesis [91].

Following this, the pro-inflammatory M1-like polarization state changes towards an alternatively activated regenerative anti-inflammatory M2-like polarized phenotype, marking the beginning of the subsequent tissue restoration phase to resolve the inflammatory process [92,93,94]. In contrast to M1-like polarized macrophages, M2-like polarized macrophages play a crucial role in initiating matrix remodeling; while the M1-like polarized phenotype can only release an inactive complexed form of the matrix protease MMP9, M2-like polarized macrophages release an active form of MMP9 [95]. Consequently, the anti-inflammatory M2-like polarized phenotype mirrors the classical pro-angiogenic polarization state correlating with matrix remodeling, resolution of inflammation, and tissue repair [47,96].

Previous studies of our own group showed that administration of recombinant CIRP induced the expression of M1-like but not M2-like polarization markers on macrophages in vitro. Moreover, we found a predominance of M2-like polarized macrophages in ischemic muscle tissue in CIRP-deficient mice without any altered general macrophage accumulation compared to wild-type control mice [59]. In the present study, we observed similar findings, i.e., a significant increase in M2-like and a significant reduction in M1-like polarized macrophages with no changes in the total number of macrophages accumulated in ischemic muscle tissue of eCIRP-depleted mice compared to control mice. The increased number of M2-like polarized macrophages in mice that underwent eCIRP depletion possibly indicates that these mice—in contrast to control mice that still show a high number of infiltrating M1-like polarized macrophages—have already moved from the initial inflammatory phase towards the tissue regenerating phase.

In accordance with our in vitro results along with published information, the high number of M1-like polarized macrophages in control mice may be related to three different responses, triggered by eCIRP: (a) eCIRP promotes the expression of pro-inflammatory M1-like polarization markers in macrophages; (b) eCIRP strongly induces the expression of the pro-inflammatory genes IL-6 and MCP-1 in microvascular endothelial cells. The latter reaction will result in increased immune cell migration and infiltration, especially for monocytes, to the site of inflammation [97]. Moreover, since a deficiency of MCP-1 was described to enhance M2-like polarization in macrophages [98], a reduced expression of MCP-1 in mice lacking eCIRP could lead to a reduced induction of pro-inflammatory M1-like polarized macrophages in eCIRP-deficient mice. Also, an eCIRP-dependent release of pro-inflammatory chemokines and cytokines from a wide range of cells has been described [63]. Thus, a pro-inflammatory environment, induced by eCIRP´s propensity as a DAMP, is likely to promote the pro-inflammatory M1-like polarization status in macrophages and could lead to the observed high number of neutrophils, NETs, and the leukocyte infiltration at the site of ischemic muscle tissue. (c) As already mentioned above, eCIRP induces a particular subtype of NETs that are described to impair macrophage efferocytosis and thus may prolong the inflammatory phase. Therefore, the decreased amount of phagocyting M1-like polarized macrophages in combination with decreased apoptotic areas in eCIRP-blocked mice could reflect an improved macrophage efferocytosis and consequently an enhanced transition from the inflammatory to the tissue restitution phase.

Recently, a protective effect in ischemic stroke was reported for an eCIRP-derived peptide, which interferes with eCIRP´s binding ability to the MD2 receptor, resulting in a significant reduction of the ischemic infarct area as well as the inhibition of apoptosis and necroptosis in murine and rhesus monkey models [99]. This experimental approach highlights an important milestone in designing CIRP-related therapeutic treatments for ischemia-related pathologies.

With the present study, we demonstrated that several observations regarding angiogenesis and the associated leukocyte recruitment in CIRP-deficient mice also hold true for eCIRP-blocked mice. Thus, we propose that the lack of CIRP´s extracellular properties, especially as an inflammatory DAMP, are mainly responsible for the increased angiogenic process we have found in CIRP-deficient mice. Taken together, the administration of eCIRP-depleting drugs may be a valuable approach to modulate inflammatory processes and to improve angiogenesis in ischemic muscle tissue via the induction of M2-like macrophage polarization.

## 4. Materials and Methods

### 4.1. Animals and Treatments

All experimental setups were permitted by the Bavarian Animal Care and Use Committee (ethical approval code: ROB-55.2Vet-2532.Vet_02-17-99, approved on 8 December 2017) and were conducted in strict accordance with the German animal legislation guidelines. Mice were fed a standard laboratory diet and were housed in a temperature-controlled room on a 12 h light–dark cycle. For all experiments, adult male SV-129 (Charles River Laboratories, Sulzfeld, Germany) mice, aged 8–12 weeks, were sacrificed at 24 h or 7 days (*n* = 5 per group) after the surgical procedure. 30 minutes prior to surgical intervention, mice were either treated i.v. with a neutralizing anti-CIRP antibody (Abcam, ab106230, Cambridge, UK, 1 mg/kg), an isotype antibody (Abcam, ab37373, 1 mg/kg), or phosphate-buffered saline (PBS, PAN Biotech, Aidenbach, Germany, pH 7.4, 1 mL/kg) and then two, four and six days after surgery. To ascertain the proliferation rate of endothelial cells in the lower hindlimb 7 days after surgery, SV-129 mice were daily injected with 100 µL BrdU (bromodeoxyuridine) (Sigma-Aldrich, St. Louis, MO, USA) (12.5 mg/mL BrdU in PBS) i.p., starting directly after the surgical intervention.

### 4.2. Femoral Artery Ligation and Tissue Processing

To promote angiogenesis in the gastrocnemius muscle of the lower hindlimb, unilateral femoral artery ligation (FAL) was performed on the right femoral artery while the left artery was sham-operated and served as an internal control, as previously described [60]. Twenty minutes in advance of the surgical procedure, mice were anesthetized with a combination of fentanyl (0.05 mg/kg, CuraMED Pharma, Karlsruhe, Germany), midazolam (5.0 mg/kg, Ratiopharm GmbH, Ulm, Germany), and medetomidine (0.5 mg/kg, Pfister Pharma, Berlin, Germany). Prior to tissue sampling, 24 h or 7 days after FAL, mice were again anesthetized as described above. For tissue collection, the hindlimbs were perfused with adenosine buffer (1% adenosine (Sigma-Aldrich), 5% bovine serum albumin (BSA, Sigma-Aldrich), dissolved in PBS) and 3% paraformaldehyde (PFA, Merck, Darmstadt, Germany, dissolved in PBS). For immunohistology, both gastrocnemius muscles of each mouse were collected subsequently to perfusion, embedded in Tissue-Tek compound (Sakura Finetek Germany GmbH, Staufen, Germany), and cryopreserved at −80 °C.

### 4.3. Immunohistology

The cryopreserved gastrocnemius muscles from 24 h and 7 days after FAL were cut in 10 µm thick slices. For BrdU-staining, 1 N HCl was added to the cryosections in a humidified chamber at 37 °C for 30 min, followed by permeabilization with 0.2% Triton X-100 solution (AppliChem GmbH, Darmstadt, Germany) in 1 × PBS/0.1% Tween-20 (AppliChem GmbH)/0.5% BSA for 2 min, then blocked with 10% goat serum (Abcam, ab7481, Cambridge, UK) in 1 × PBS/0.1% Tween-20/0.5% BSA) for 1 h at room temperature (RT), and subsequently incubated with the primary anti-BrdU-antibody (Abcam, ab6326, dilution 1:50 in 10% goat serum) at 4 °C overnight. For secondary staining, the cryosections were treated with a goat anti-rat Alexa Fluor^®^-546 antibody (Invitrogen, Thermo Fischer Scientific, A-11081, Carlsbad, CA, USA, dilution 1:100) for 1 h at RT. Following secondary blocking with 1 × PBS/0.1% Tween-20/4% BSA for 30 min at RT, the sections were incubated with an anti-CD31-Alexa Fluor^®^ 647 antibody (Biolegend, 102516, San Diego, CA, USA, dilution 1:50 in 1 × PBS/0.1% Tween-20) applied to label endothelial cells, together with an anti-CD45-Alexa Fluor^®^ 488 antibody anti-CD45-Alexa Fluor^®^ 488 antibody (BioLegend, 11-0451-85, dilution 1:100 in 1 × PBS/0.1% Tween-20) implemented as a pan-leukocyte marker for 2 h at RT.

Macrophages were stained with an anti-CD68-Alexa Fluor^®^ 488 antibody (Abcam, ab201844, dilution 1:200 in PBS), which was co-incubated with an anti-MRC1 antibody (Abcam, ab64693, dilution 1:200 in PBS) as a macrophage polarization marker, at 4 °C overnight. Secondary antibody staining was performed with a donkey-anti-rabbit Alexa Fluor^®^ 546 (Invitrogen, A-10040) for 1 h at RT.

To label NETs in tissue collected 24 h after FAL, cryosections were firstly permeabilized with 0.2% Triton X-100 solution in 1 × PBS/0.1% Tween-20/0.5% BSA for 2 min, followed by blocking with 10% donkey serum (Abcam, ab7475) in 1 × PBS/0.1% Tween-20/0.5% BSA for 1 h at RT and then incubated with the primary antibodies anti-myeloperoxidase (MPO; R&D Systems, AF3667, Minneapolis, MN, USA, dilution 1:20 in 10% donkey serum in 1 × PBS/0.1% Tween-20/0.5% BSA) and anti-citrullinated histone H3 antibody (Cit-H3; polyclonal rabbit anti-Histone H3 (citrulline R2 + R8 + R17), Abcam, ab5103, dilution 1:100 in 10% donkey serum in 1 × PBS/0.1% Tween-20/0.5% BSA) at 4 °C overnight. Secondary antibody staining was performed with a donkey anti-goat Alexa Fluor^®^ 594 (Invitrogen, A-11058, dilution 1:100 in 1 × PBS/0.1% Tween-20) and a donkey anti-rabbit Alexa Fluor^®^ 488 antibody (Invitrogen, A-21206, dilution 1:200 in 1 × PBS/0.1% Tween-20) for 1 h at RT.

To quantify apoptotic cells throughout the gastrocnemius muscle sections, we used an ApopTag^®^ Plus Fluorescein in Situ Apoptosis Detection Kit (EMD Millipore Corp., Burlington, MA, USA) according to the manufacturers’ instruction. Additionally, all cryosections were counter-stained with DAPI (Thermo Fisher Scientific, 62248, dilution 1:1000 in PBS) for labeling of nucleic DNA for 10 min at RT.

An antifade mounting medium (Dako, Agilent, Santa Clara, CA, USA) was applied to mount the stained tissue samples. Gastrocnemius cryosections from ischemic (occluded) and non-ischemic (sham-operated) side harvested 24 h after FAL was used for neutrophil and NETs labeling, while tissue collected 7 days after FAL were stained for capillaries, leukocytes, macrophages, and apoptotic cells.

For microscopic analysis, we used a confocal laser scanning microscope LSM 880 (Carl-Zeiss Jena GmbH, Jena, Germany) with a 20× objective (415 µm × 415 µm) as well as an epifluorescence microscope (Leica DM6 B, Leica microsystems, Wetzlar, Germany) with a 20× objective (630 µm × 475µm). For each muscle section, we analyzed 5 defined fields of view to count cells, muscle fibers, and NETs. To ascertain the areas of apoptotic cells (%), the total gastrocnemius muscle area and the apoptotic cell areas were measured and compared. CD31/CD45/BrdU/DAPI and apoptosis staining were investigated with the epifluorescence microscope. CD68/MRC1/DAPI and MPO/CitH3/DAPI stains were analyzed with the confocal laser scanning microscope. Cell counting, as well as analysis of the apoptotic muscle area, were conducted using ImageJ software with the Cell Counting and Region of Interest plugins. We calculated the capillary (CD31^+^/CD45^−^ cells were considered endothelial cells) per muscle fiber ratio as described before to evaluate the processes of angiogenesis [61].

### 4.4. Cell Culture and Proliferation Assays

The myocardial endothelial MyEnd cell line was grown in Dulbecco’s modified Eagle medium (DMEM, Gibco, Darmstadt, Germany) with 10% fetal calf serum (FCS) and 1% penicillin/streptomycin (100 U/mL and 100 mg/mL, Sigma-Aldrich). The MyEnd cells showed typical endothelial properties and, as they grew to complete confluence, were highly positive for the endothelial marker CD31 [100].

The viability of cells was determined using the CellTiter 96^TM^ non-radioactive cell proliferation assay from Promega (Mannheim, Germany). The cells were seeded on 96-well culture plates and cultured for 4 h. Prior to stimulation, cells were washed once with phosphate-buffered saline (PBS) and incubated in serum-free cell culture medium containing different concentrations of recombinant murine CIRP (Hölzel Diagnostika, Köln, Germany). After 24 h, one solution reagent from Promega was added and the amount of the formazan product was measured by its absorbance at 500 nm, which corresponds to the number of viable cells. Absorbance measured in the absence of rmCIRP was set to 100%.

### 4.5. Quantitative Real-Time PCR (qPCR)

Following treatment of MyEND with different concentrations of rmCIRP as indicated in the legends of the corresponding figure, cells were washed twice with PBS, lysed, and RNA was isolated with the total RNA extraction kit (Peqlab). For qPCR analysis, 1 µg of RNA was reverse-transcribed using the High-Capacity cDNA Reverse Transcription Kit (Applied Biosystems, Carlsbad, CA, USA) and DNA amplification was performed with a StepOne Plus cycler (Applied Biosystems) in a reaction volume of 10 µL using the SensiMix Sybr Kit (Bioline, Luckenwalde, Germany) with 50 pmol of each primer. To avoid the amplification of genomic DNA, primers were designed to span exon-exon junctions. The qPCR was performed under the following conditions: an initial denaturation step at 95 °C for 8.5 min followed by 45 cycles, consisting of denaturation (95 °C, 30 s), annealing (60 °C, 30 s) and elongation (72 °C, 30 s). Melt curve analysis was performed to control specific amplification. Results were normalized to the expression levels (E) of actin and expressed as the ratio of E(target)/E(Actin). The following mouse primers were used: IL-6 forward 5′-CTCTGCAAGAGACTTCCATCCA-3′; IL-6 reverse 5′-TTGGAAGTAGGGAAGGCCG-3′; MCP-1 forward 5′-AAGCTGTAGTTTTTGTCA CCAAGC-3′; MCP-1 reverse 5′-GACCTTAGGGCAGATGCAGTT-3′; CIRP forward 5′-CTACTATGCCAGCCGGAGTC; CIRP reverse 5′-GCTCTGAGGACACAAGGGTT-3′; ß-actin forward 5′-CGCGAGCACAGCTTCTTTG-3′; ß-actin reverse 5′-CGTCATCCAT GGCGAACTGG-3′.

### 4.6. Statistical Analysis

Statistical analyses were carried out and graphically plotted with GraphPad Prism 8 (GraphPad Software, La Jolla, CA, USA). Data are means ± standard error of the mean (S.E.M.). Statistical analyses were performed by using the one-way analysis of variance (ANOVA) with the Tukey’s multiple comparisons test. The findings were considered statistically significant at *p <* 0.05.

### 4.7. Illustration

The illustration from Figure 6 was designed and rendered with BioRender.com.

## Data Availability

The data presented in this study is available on request from the first author.

## Supplementary Materials

The following are available online at https://www.mdpi.com/article/10.3390/ijms22179484/s1.
