# Supplementary material for: The Absence of Extracellular Cold-Inducible RNA-Binding Protein (eCIRP) Promotes Pro-Angiogenic Microenvironmental Conditions and Angiogenesis in Muscle Tissue Ischemia
Source: Int J Mol Sci. 2021 Aug 31;22(17):9484. doi: 10.3390/ijms22179484 (PMC8431021; doi:10.3390/ijms22179484)
Supplement: Supplementary file 1 [file ijms-22-09484-s001.zip › ijms-1323177-supplementary.pdf]

International Journal of *Molecular Sciences*

MDPI

Article

The Absence of Extracellular Cold-Inducible RNA-Binding Protein (eCIRP) Promotes Angiogenic Pro-Angiogenic Inflammatory Microenvironmental Conditions and Angiogenesis in Muscle Tissue Ischemia

Matthias Kübler[1,2,], Sebastian Beck[1,2,], Lisa Lilian Peffenkköver[3], Philipp Götz[1,2], Hellen Ishikawa-Ankerhold[1,4,], Klaus T. Preissner[3], Silvia Fischer[3], Manuel Lasch[1,2,5], Elisabeth Deindl[1,2,*]

[1] Walter-Brendel-Centre of Experimental Medicine, University Hospital, Ludwig-Maximilians-Universität München, 81377, Munich, Germany; Matthias.Kuebler@med.uni-muenchen.de (M.K.); sebastian.beck@med.uni-muenchen.de (S.B.); P.Goetz@med.uni-muenchen.de (P.G.); Hellen.Ishikawa-Ankerhold@med.uni-muenchen.de (H.I.-A.); manuel_lasch@gmx.de (M.L.); Elisabeth.Deindl@med.uni-muenchen.de (E.D.)

[2] Biomedical Center, Institute of Cardiovascular Physiology and Pathophysiology, Ludwig-Maximilians-Universität München, 82152, Planegg-Martinsried, Germany

[3] Department of Biochemistry, Faculty of Medicine, Justus Liebig University, 35392, Giessen, Germany; Lilli.Peffenkoever@gmail.com (L.L.P.), Klaus.T.Preissner@biochemie.med.uni-giessen.de (K.T.P.) Silvia.Fischer@biochemie.med.uni-giessen.de (S.F.)

[4] Department of Internal Medicine I, Faculty of Medicine, University Hospital, Ludwig-Maximilians-Universität München, 81377, Munich, Germany

[5] Department of Otorhinolaryngology, Head and Neck Surgery, University Hospital, Ludwig-Maximilians-Universität München, 81377, Munich, Germany

* Correspondence: Elisabeth.Deindl@med.uni-muenchen.de; Tel.: +49 (0) 89 2180 76504

Supplement

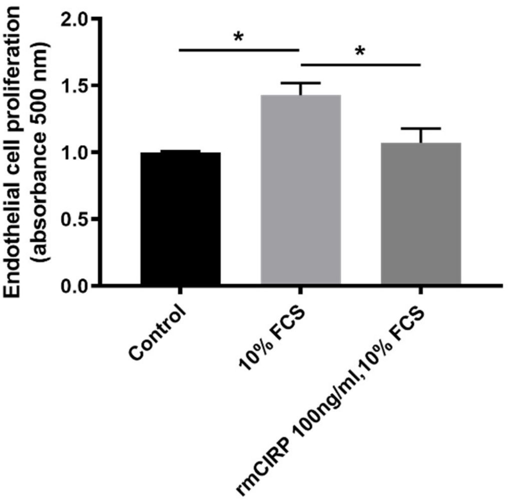

Figure S1. Effects of Cold-inducible RNA-binding protein (CIRP) on the proliferation of microvascular endothelial cells. Endothelial cells (MyEnd cells) were cultivated with either serum-free (control) or with 10% FCS in the absence or presence of recombinant murine CIRP (rmCIRP) (100ng/ml). After 24 h, one solution reagent from Promega was added and the amount of the formazan product was measured by its absorbance at 500nm, which corresponds to the number of viable cells. Data are means ± S.E.M., n = 3-5, * $p < 0.05$ determined by one-way ANOVA with the Tukey's multiple comparisons test.

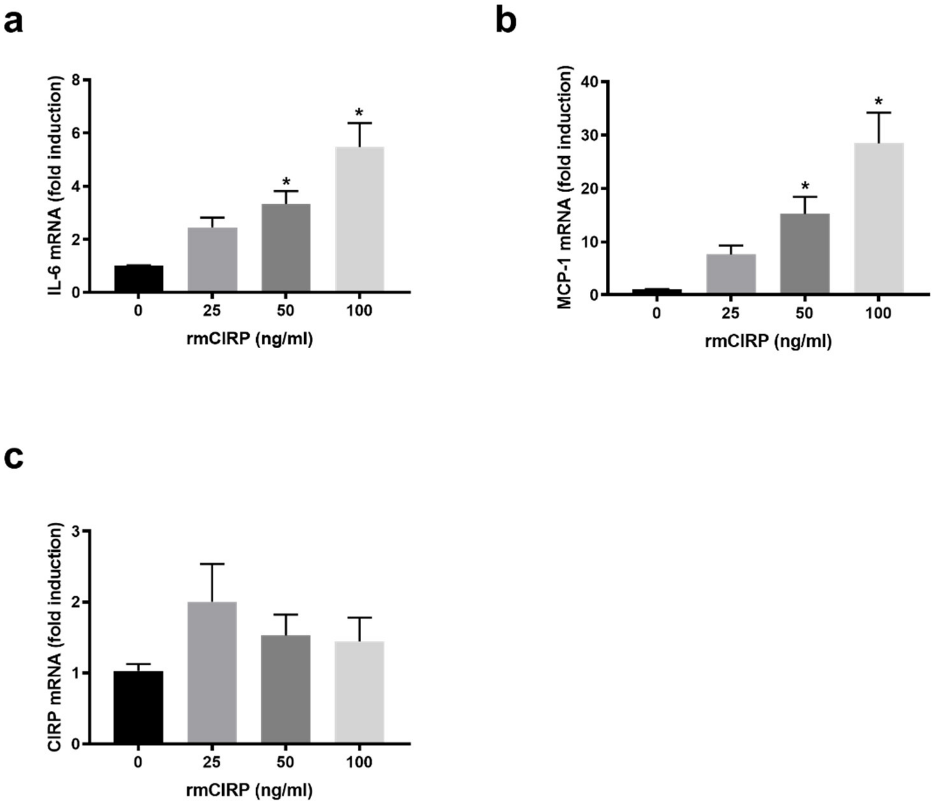

Figure S2. Inflammatory activities of Cold-inducible RNA-binding protein (CIRP) in endothelial cells. Endothelial cells (MyEnd cells) were treated for 4 h with different concentrations of recombinant murine CIRP (rmCIRP). Untreated cells served as control. Real-time RT-PCR was used to determine transcript levels of interleukin 6 (IL-6) (**a**), monocyte chemoattractant protein-1 (MCP-1) (**b**), or CIRP (**c**). Data are means ± S.E.M., n = 3-5, * $p < 0.05$ determined by one-way ANOVA with the Tukey's multiple comparisons test.
